# Pharmacological Treatments and Natural Biocompounds in Weight Management

**DOI:** 10.3390/ph16020212

**Published:** 2023-01-30

**Authors:** Amin Gasmi, Pavan Kumar Mujawdiya, Amine Nehaoua, Mariia Shanaida, Yuliya Semenova, Salva Piscopo, Alain Menzel, Volodymyr Voloshyn, Olena Voloshyn, Volodymyr Shanaida, Geir Bjørklund

**Affiliations:** 1 Société Francophone de Nutrithérapie et de Nutrigénétique Appliquée, 69100 Villeurbanne, France; 2 Research Department, Precision Health International, Ain Oulmene 19200, Algeria; 3 Inochi Care Private Limited, New Delhi 110017, India; 4 Laboratory of Physical Activity Sciences and Public Health, Mohamed Lamine Debaghine Sétif 2 University, Setif 19000, Algeria; 5 I. Horbachevsky Ternopil National Medical University, 46001 Ternopil, Ukraine; 6 Nazarbayev University School of Medicine, Astana 010000, Kazakhstan; 7 Department of Nutritional Research and Development, Nutri-Logics SA, 9990 Weiswampach, Luxembourg; 8 Laboratoires Réunis, 6131 Junglinster, Luxembourg; 9 Ternopil Volodymyr Hnatiuk National Pedagogical University, 46027 Ternopil, Ukraine; 10 Ternopil Ivan Puluj National Technical University, 46001 Ternopil, Ukraine; 11 Council for Nutritional and Environmental Medicine (CONEM), 8610 Mo i Rana, Norway

**Keywords:** obesity, metabolic syndrome, synthetic drugs, natural compounds

## Abstract

The obesity pandemic is one of society’s most urgent public health concerns. One-third of the global adult population may fall under obese or overweight by 2025, suggesting a rising demand for medical care and an exorbitant cost of healthcare expenditure in the coming years. Generally, the treatment strategy for obese patients is largely patient-centric and needs dietary, behavioral, pharmacological, and sometimes even surgical interventions. Given that obesity cases are rising in adults and children and lifestyle modifications have failed to produce the desired results, the need for medical therapy adjunct to lifestyle modifications is vital for better managing obesity. Most existing or past drugs for obesity treatment target satiety or monoamine pathways and induce a feeling of fullness in patients, while drugs such as orlistat are targeted against intestinal lipases. However, many medications targeted against neurotransmitters showed adverse events in patients, thus being withdrawn from the market. Alternatively, the combination of some drugs has been successfully tested in obesity management. However, the demand for novel, safer, and more efficacious pharmaceutical medicines for weight management does exist. The present review elucidates the current understanding of the available anti-obesity medicines of synthetic and natural origin, their main mechanisms of action, and the shortcomings associated with current weight management drugs.

## 1. Introduction

Obesity, a metabolic complication, was initially considered a disease of positive energy balance due to overeating and a sedentary lifestyle. This perception led to the belief that dietary and short-term pharmacological interventions can easily control the obesity pandemic [1]. However, the scientific community in 1985 recognized obesity as a chronic disorder. The first approved class of drugs for obesity was amphetamines, which were subsequently removed from the market due to addiction and adverse events associated with the long-term use of amphetamines. This highlighted the need to design and develop safer alternatives for long-term use in obesity management [2]. The treatment of obesity is highly patient-centric, and various pharmacological and surgical approaches may require depending on the affected individuals. Effective strategies for treating obesity involve lifestyle intervention, complementary medicine, and alternative therapy, including drug treatment or bariatric surgery [1]. Thus, acupuncture is a good example of an effectively used alternative therapy in obesity coping due to its positive effect on hypothalamus functioning [3].

Considering the chronic nature of obesity, a three-step weight management practice is recommended. The first stage involves the use of dietary intervention, alteration in lifestyle and behavior, and increased physical activity [1]. For instance, a low-fat and high-protein diet in the Mediterranean region could help achieve weight loss and prevent muscle loss and osteoporosis [4]. Maintaining a balanced diet with a sufficient consumption of vitamins and trace elements and limiting the consumption of high-calorie foods, along with the proper intake of drinking water and physical activity, can prevent the development of metabolic syndrome and adiposity [5,6]. In the second stage, pharmacological treatment options are recommended alongside lifestyle modifications. Drug therapy is considered the most effective way to lose weight [1]. In the final step, surgical procedures such as bariatric surgery are recommended for obese patients who are unresponsive to non-surgical intervention and have uncontrolled and morbid obesity [7].

The pharmacological treatment for obesity becomes necessary given that the latest data from the World Obesity Federation suggests that 2.7 billion adults will fall under the obese category by 2025; thus, a huge demand for medical care and therapeutic interventions will arise in the near future. The rising global epidemic will result in huge medical costs pegged at USD 1.2 trillion per annum unless various interventions control the epidemic. Several anti-obesity drugs have been approved in the past. For instance, phentermine, diethylpropion, rimonabant, taranabant, sibutramine, orlistat, lorcaserin, and tesofensine are some of the anti-obesity medications for weight management [8]. Several anti-obesity drugs target monoamine neurotransmitter pathways, producing a feeling of fullness [9]. However, several anti-obesity drugs targeted towards neurotransmitters were withdrawn due to adverse psychiatric side effects and thus should not be prescribed for obese patients with psychological disorders [10]. Importantly, very limited behavioral data are available for existing anti-obesity drugs, making it essential to collect behavioral data during the early stages of drug development. Moreover, a lack of understanding of the molecular, cellular, and physiological targets of anti-obesity drugs may hamper the progress of drug development and consequently delay finding novel and effective anti-obesity molecules [11].

The present review elucidates the current understanding of the available anti-obesity drugs of synthetic and natural origin, their main mechanisms of action, and the shortcomings associated with current weight management medicines.

## 2. Drugs-Induced Obesity

Weight gain is one of the most commonly observed side effects of drugs used in the treatment/management of a few disorders. Some medicines can lead to weight gain as high as 10% of the total body weight and thus increases the health risk of individuals on medications. Since weight gain is a common underlying cause of several metabolic disorders, strict and regular monitoring of the patients for any adverse outcomes associated with the drug treatment is required [12]. For instance, glucocorticoids and anti-HIV (Human Immunodeficiency Virus) drugs are associated with weight gain and lipodystrophy. Similarly, anti-sense apo-B oligonucleotides and MTTP (Microsomal triglyceride transfer protein) inhibitors employed in managing lipid disorders can cause the ectopic deposition of fat, especially in the liver, and changes the fat distribution in the body [12]. It is well established that antidepressants, atypical antipsychotics, and mood-stabilizing agents induce weight gain by increasing appetite [13]. A UK study in 136,762 men and 157,957 women demonstrated that the use of antidepressants was associated with weight gain at the population level and suggested that the risk for weight gain must be considered during the antidepressant treatment [14]. Olanzapine, one of the most efficacious drugs for schizophrenia, is associated with significant weight gain and metabolic dysfunctions such as insulin insensitivity. A recent study has shown that ALKS 3831, a combination of Olanzapine and Samidorphan (an opioid receptor agonist), can reduce weight gain and metabolic complications associated with olanzapine [15]. In another observation, the weight gain induced by second-generation antipsychotics, which include aripiprazole, olanzapine, risperidone, and clozapine, can be controlled by metformin [16]. Pisano et al. [17] demonstrated that youths taking antipsychotics show significantly higher levels of C-peptide, glucose-dependent insulinotropic polypeptide, and adipsin, indicating β-cell stress and higher risk for insulin resistance in youths showing antipsychotic-induced weight gain. Thus, the use of medications that cause weight gain requires extensive monitoring to avoid adverse metabolic complications of the drugs. It is pertinent to mention that the fat reserved (subcutaneous or visceral) affected by the drug must also be considered while considering the side effects of the drug. For example, oral antidiabetic drugs thiazolidinediones (TZDs) are insulin sensitizers and commonly prescribed for glycemic control. Interestingly, TZD-induced weight gain is limited to subcutaneous adipose tissue only, and TZDs do not alter the fat deposition in visceral tissues. This suggests that TZD-induced weight gain is less likely to induce insulin resistance and other metabolic complications associated with increased visceral fat mass [18].

A recent study [19] showed that the active use of antidepressants in therapy continues and even grows in England, despite controversy about the effectiveness of these drugs and the potential harm to the patient if use is discontinued. Attention is drawn to the fact that one of the side effects of antidepressant therapy is weight increase [19]. Selective serotonin reuptake inhibitors are often used to treat depression. However, research shows that using these drugs leads to weight growth [20]. Patients taking antipsychotic drugs—in particular, olanzapine—face the problem of metabolic disorders and significant weight gain. In this regard, a study on the effect of miricorilant, a selective glucocorticoid receptor modulator, on olanzapine-associated weight gain suggested that miricorilant could potentially be an option to mitigate the harmful effects of olanzapine. Still, research needs to be continued [21]. New research confirms that glucocorticoid therapy can cause several negative effects, including weight gain, dyslipidemia, and hyperglycemia [22]. Long-term studies of obese patients have shown that their cortisol values are in a high physiological range. Elevated levels of glucocorticoids are directly related to weight gain. Therefore, it is advisable to analyze the mechanisms of this relationship to develop a therapeutic correction to achieve sustainable weight loss [23].

## 3. Novel Drugs in Obesity Treatment

Obesity is a complex metabolic disorder affecting several body organs and tissues. Obesity severely disturbs the metabolic processes in the system and causes hyperglycemia, impaired glucose tolerance, dyslipidemia, and gastrointestinal abnormalities [24]. Currently, obesity is managed with the help of anti-obesity or weight loss medications that help reduce body weight gain. The US Food and Drug Administration (FDA) has currently approved five drugs: orlistat (Xenical and Alli), lorcaserin (Belviq), phentermine-topiramate (Qsymia), naltrexone-bupropion (Contrave), and liraglutide (Saxenda) for the treatment of obesity. As per the FDA guidelines, an obese individual can take anti-obesity drugs as long as they cause benefits without any unpleasant side effects. Importantly, anti-obesity medications must not be taken by pregnant women or women who are planning pregnancy, as they may harm the fetus. Moreover, using anti-obesity medications in obese subjects can also cause side effects such as oily stool, incontinence, headaches, gastrointestinal upset, acute pancreatitis, nausea, and fecal urgency [25]. The present section explores some commonly prescribed anti-obesity drugs and the clinical evidence supporting their usefulness in managing obesity.

Although researchers point to significant progress in the treatment of several pathologies caused by being overweight, the treatment of obesity itself remains currently problematic. Anti-obesity drugs often do not give the expected effect and have an insufficient level of safety. Recent advances in the analysis of the molecular connection between the brain and the intestine will help create a new generation of anti-obesity drugs that can provide stable weight loss for the patient [26]. The old weight control medications contributed to only minor additional weight loss. The modern drug semaglutide makes possible a much greater loss of body weight—an average of 15% weight loss in 1 year. This efficacy of semaglutide can potentially increase the use of pharmacotherapy by clinicians to correct patient weight. A positive result from a cardiovascular outcome study to test the effectiveness of an anti-obesity drug would further the importance of weight management in controlling cardiometabolic disease [27]. Similar optimistic conclusions in the treatment of overweight were obtained in the study of the effectiveness of phentermine-topiramate and GLP-1 receptor agonists [28].

The anti-obesity drugs have been well tolerated in randomized controlled experiences, although the findings may need to be more generalizable to clinical practice. Studies have also shown that anti-obesity medications did not cause severe complications but only some mild to moderate ones. However, despite the effectiveness of anti-obesity drugs, they need to be more evidence-based [29].

### 3.1. Orlistat

Orlistat is one of the most commonly prescribed anti-obesity drugs sold under the trade name Xenical or Alli. Additionally, known as tetrahydrolipstatin, orlistat is a saturated derivative of lipstatin, a natural molecule isolated from Streptomyces toxytricini. Like lipstatin, Orlistat also irreversibly inhibits the activity of pancreatic and gastric lipases. These lipases convert dietary fats into free fatty acids, thus making them essential for digestion and the subsequent absorption of dietary fats. Orlistat inhibits the activity of lipases by binding with the enzyme’s serine residue, which inhibits the hydrolysis of triglycerides into free fatty acids. This undigested fat is secreted into the feces, thus reducing the overall calorie intake of an obese patient. Importantly, the inhibitory activity of orlistat is specific to only lipases, and it does not inhibit the activity of other digestive enzymes such as trypsin, amylase, chymotrypsin, and phospholipases [30]. Since orlistat inhibits fat absorption, it inhibits the absorption of fat-soluble vitamins such as vitamins A, D, E, and K. Hence, patients taking orlistat therapy may need vitamin supplements. Orlistat is generally prescribed with a mildly hypocaloric diet. Per the guidelines, orlistat treatment must be prescribed only for obese patients who have lost at least 2.5 kg weight in 4 weeks with the help of dietary interventions alone.

Moreover, orlistat is not recommended for patients who lose less than 5% of body weight during a 12-week treatment cycle. Finally, orlistat must be discontinued in cases where weight loss is less than 5% of the initial weight after 12 weeks of treatment. As per the European guidelines, the duration of orlistat treatment should be, at most, two years [31].

However, studies have shown that weight loss due to orlistat may not always be substantial; a variability in weight loss has been observed, with several patients showing no weight loss at all. Additionally, there is a high attrition rate associated with orlistat use due to several unpleasant side effects associated with its use [32]. A study with 500 subjects showed that treatment with orlistat (*n* = 400) and liraglutide (*n* = 100) during a 7-month follow-up study significantly reduced body weight, helped manage the plasma glucose and lipid profiles, and reduced the systolic blood pressure. However, patients taking liraglutide lost significantly more weight (−7.7 kg) than the orlistat group (−3.3 kg) [33]. It has been observed that orlistat acts via multiple pathways that include lipase inhibition, the modulation of neurotransmitters such as glutamate and dopamine levels, and elevation of the glycogen levels [34].

Despite active research aimed at creating drugs for correcting and controlling body weight, orlistat is one of the few approved in Germany for treating obesity [35]. Obesity increases the risk of hyperuricemia, which contributes to the development of gout and cardiovascular disease. Currently, scientists continue to conduct active orlistat research on certain aspects of its use in treating ocular, which concerns monitoring issues, possible side effects, pharmacodynamics, pharmacokinetics, and related interactions [36]. There is new information about creating an oral capsule with a modified release of orlistat and acarbose (MR-OA). All doses tested were safe and well tolerated, with no serious side effects. The delay in increasing the concentration of orlistat in plasma indicates the effectiveness of the properties of the modified release of the MR-OA composition [37]. A separate area of research is now the analysis of the effect of orlistat on uric acid in blood serum in adults [38].

### 3.2. Liraglutide 3.0

Liraglutide 3.0 is a recently approved anti-obesity drug marketed under the trade names Saxenda^®®^ (Novo Nordisk, Copenhagen, Denmark) and Victoza^®®^ (Novo Nordisk, Copenhagen, Denmark). In 2010, the FDA approved a 1.8 mg daily subcutaneous injection of Liraglutide as an adjunct therapy for managing type 2 diabetes mellitus. However, recently, the FDA approved a daily dose of 3 mg of Liraglutide for chronic weight management in obese individuals with a Body Mass Index (BMI) ≥ 27 kg/m^2^ and suffering from other comorbidities [39]. Liraglutide 3.0 has been clinically evaluated for its efficacy in weight management. A randomized control trial in 198 patients showed that Liraglutide 3.0, in combination with intensive behavioral therapy, helped obese patients lose significantly more weight (−5.8%) compared with the placebo group (−1.5%). Moreover, 51.8% of participants on Liraglutide achieved a ≥5% reduction in weight compared with the placebo group (24%). Liraglutide 3.0 mg treated patients showing significantly lower glycated hemoglobin levels and glucose values than the placebo group. No safety and tolerability issues were observed in the Liraglutide-treated group [40]. In a 56-week clinical trial, obese patients receiving Liraglutide lost 8.4 ± 7.3 kg body weight, while the placebo-treated group lost only 2.8 ± 6.5 kg. Moreover, 63.2% of patients in the Liraglutide-treated group showed at least 5% loss in body weight, while, in the placebo group, only 27.1% of subjects lost at least 5% weight. Similarly, more patients in the Liraglutide group showed >10% loss in body weight than in the placebo group (33.1% vs. 10.6%). However, Liraglutide was associated with side effects such as diarrhea or nausea [41]. In another interventional study, obese adults prescribed a combination of Liraglutide 3.0 and lifestyle therapy lost significantly more weight than the placebo and lifestyle therapy group. However, more participants taking Liraglutide displayed adverse gastrointestinal effects, leading to the trial treatment discontinuation [42]. At the molecular level, Liraglutide acts by reducing appetite and lowering energy intake, and these actions of Liraglutide are independent of the hypoglycemia effects. Interestingly, Liraglutide also reduces the risk of cardiovascular disorders in subjects with type 2 diabetes mellitus. However, its cost and requirement for daily injections are some limiting factors in its use [43].

Modern studies have confirmed the positive effect of Liraglutide in treating patients who have regained weight after bariatric surgery [44]. Recent studies confirm the positive effect of Liraglutide 3.0 mg on weight loss in patients and indicate a positive effect of the drug on the metabolism of myocardial cells [45]. New findings indicate the efficacy of Liraglutide 3.0 mg subcutaneously in obese patients [46]. There is further information on significant weight loss in patients treated with Liraglutide during an initial 4-month period [47].

### 3.3. Phentermine/Topiramate

A combination of phentermine and topiramate (delayed-release) has been sold for treating obesity under the brand name Qsymia in the United States since September 2012. Phentermine is recommended for short-term weight loss due to its anorexigenic properties, and topiramate is primarily recommended to prevent seizures and migraine. However, a combination of these two drugs synergistically reduced weight gain, and the weight loss achieved by the fixed-dose combination was higher than the weight loss achieved when either drug was given alone. At the molecular level, phentermine promotes weight loss by reducing food intake by enhancing the release of the neurotransmitter norepinephrine and possibly blocking its reuptake, thus increasing its levels. Topiramate is derived from fructose, an FDA-approved drug for treating seizure disorders (400 mg/day) or migraines (100 mg/day). Several clinical trials have demonstrated that a fixed-dose combination of phentermine and topiramate helped patients achieve a sustained and robust weight loss maintained for up to 2 years in more than 50% of the study participants [48]. In a 28-week, randomized, controlled trial, Aronne et al. [49] found that the combination of PHEN/TPM ER (phentermine/topiramate extended-release) was more effective in weight loss than in the placebo group and groups that received monotherapies. For instance, only 15.5% of subjects from the placebo group achieved ≥5% weight loss, while 62.1% and 66% of participants achieved ≥5% weight loss when given a fixed-dose combination of PHEN/TPM ER 7.5/46 and PHEN/TPM ER 15/92, respectively. The participants tolerated the combination well, and no serious cognitive impairment, except impairment in attention, was observed in the study participants [49]. A study involving obese adolescents between the age group of 12 and 17 years showed that a higher number of adolescents taking mid and top doses of the combination achieved ≥5% weight loss than the placebo group. The eight-week fixed-drug combination of PHEN/TPM promoted significant weight loss with no side effects and tolerability issues. The study highlighted the mid- and long-term safety profiles of PHEN/TPM in managing obesity [50]. A study involving 866 subjects showed that participants taking PHEN/TPM showed significant weight loss at the end of the 108th week. At each dose, significantly more participants achieved ≥5%, ≥10%, ≥15%, and ≥20% weight loss than the respective placebo groups, indicating the effectiveness of the combination therapy in weight management. Interestingly, the PHEN/TPM combination also improved the cardiovascular and metabolic health of the participants and lowered the incidence of diabetes in the participants [51].

Recently, phentermine/topiramate was approved in the USA for chronic weight control in pediatric patients ≥ 12 years of age in combination with increased physical activity and a low-calorie diet. In addition, phentermine/topiramate is being clinically developed in the United States for treating type 2 diabetes in obese patients and sleep apnea [52].

### 3.4. Phentermine/Diethylpropion

Diethylpropion is an amphetamine analog that suppresses appetite and has been approved as a short-term (<12 weeks) anti-obesity drug in the United States since 1959. It shows lesser effects on the central nervous system than amphetamine and, hence, has a lesser risk for drug abuse [53]. A study by Vallé-Jones et al. [54] compared the effectiveness and tolerance of phentermine and diethylpropion in obese subjects. A daily dose of 30 mg phentermine (*n* = 50) or 75 mg diethylpropion dose (*n* = 49), in combination with calorie restriction, was given to obese patients for 12 weeks. It was observed that the phentermine-treated group showed increased weight loss compared to the diethylpropion-given group [54]. In a clinical trial involving 69 obese healthy subjects on a hypocaloric diet, 50 mg of diethylpropion was given to 37 subjects for six months. The placebo group (*n* = 32) did not receive any therapeutic intervention. After a 6-month intervention, the diethylpropion group lost 9.8% of their initial body weight compared to the placebo group (3.2%). After six months, both groups received diethylpropion treatment for another six months, thus making the total study period one year.

Interestingly, the diethylpropion group given the intervention for 12 months lost 10.6% of their initial body weight. However, the placebo group that switched to diethylpropion after six months lost 7% body weight. The difference in weight loss was not significant at 12 months. Importantly, the study reported no psychiatric or cardiovascular adverse effects of diethylpropion, suggesting that its use is safe for a longer term [55]. Recently, a combination of diethylpropion and topiramate was tested on rats for anorectic effects. It was observed that a combination of lower doses of diethylpropion + topiramate synergistically increased the anorectic behavior of individual drugs without any safety concerns [56]. Interestingly, the efficacy of diethylpropion can be enhanced by carefully selecting the administration time. A recent study has demonstrated that the administration of diethylpropion to rats during their active phase promoted greater weight loss than in their inactive phase.

Moreover, diethylpropion-induced weight loss significantly improved under high-fat (HF) diet restriction compared to weight loss observed under ad libitum availability to the HF diet. The study showed for the first time that the careful selection of administration timing could significantly improve the anti-obesity properties of diethylpropion and probably for other appetite suppressants [57]. In a survey of the Mexican population, the combination of diethylpropion with diet and exercise (DEP + DaE) was more effective in weight loss than diet and exercise alone (DaE). The study concluded that DEP + DaE is a cost-effective solution for managing obesity in risk populations [58].

There are new studies on the pharmacological effects of some drugs that lead to abuse. The pharmacokinetics of cathinones was studied in experimental and prospective clinical studies. This study showed that several drugs, including diethylpropion, lead to undesirable effects that can cause dependence and abuse. The authors point to the need for future research to prevent negative manifestations in the treatment of patients [59].

### 3.5. Lorcaserin

Lorcaserin is an FDA-approved drug for the long-term management of obesity in obese individuals with BMI >30 kg/m^2^ or obese subjects with BMI > 27 kg/m^2^ and suffering from at least one obesity-associated metabolic complication. Lorcaserin is an agonist to serotonin receptors and specifically target 5HT2C receptors. Its safety and efficacy have been proven to manage obesity and related comorbidities such as cardiovascular disorders, kidney disease, and type 2 diabetes mellitus. New findings suggest that lorcaserin modulates dopaminergic pathways and helps glucose homeostasis [60]. A large clinical trial in 12,000 overweight or obese subjects with cardiovascular disease showed that 38.7% of patients (1986/5135) receiving lorcaserin (10 mg twice daily) for one year showed at least 5% weight loss in comparison with 17.4% of obese patients that received the placebo. Moreover, patients given lorcaserin showed a lower risk for cardiovascular risk factors, as evidenced by slightly better values of factors such as the lipid profile, glucose values, blood pressure, and heart rate than the placebo group. However, more patients in the lorcaserin group displayed serious hypoglycemia than in the placebo group. The study concluded that lorcaserin is a safe intervention for sustained weight loss without any adverse cardiovascular events in high-risk obese or overweight patients [61]. However, contradicting results on the efficacy of lorcaserin have been reported. It has been observed that obese patients taking lorcaserin lost only 3 kg extra weight in comparison with the placebo group. Moreover, lorcaserin-induced weight loss was not sustained, and individuals gained weight after discontinuing lorcaserin. Some commonly observed side effects of lorcaserin were dry mouth, nausea, headache, fatigue, dizziness, and euphoria. Importantly, lorcaserin increased the risk of cardiac valve disorders more than the placebo group. The clinical trials with lorcaserin were also conducted for a short duration and, hence, do not exclude the risk for various cancers such as breast cancer and astrocytoma. The use of lorcaserin to manage obesity failed to prevent obesity-associated complications and weight management. Thus, its usefulness as a weight loss drug cannot be justified [62]. It is pertinent to highlight that recent findings of a large clinical trial conducted on 12,000 subjects showed that lorcaserin increased the risk for cancer in study participants, and subjects given lorcaserin showed a higher incidence of cancer than the placebo group. Due to the increased risk of cancer with lorcaserin use, the FDA has requested drug makers to withdraw lorcaserin from the market.

New studies point to the ability of lorcaserin to inhibit glucose-stimulated insulin secretion and calcium influx in the mouse pancreatic islet. This further information on the signaling mechanism of lorcaserin is a stimulus for continued research on the functions of 5-HT2CR in β-cell biology [63]. A study was conducted on the combined treatment of lorcaserin and betahistine for cognitive dysfunction caused by obesity. The study’s results confirmed the ability of both drugs to improve cognitive function through the mechanism of action on dopaminergic signals in the prefrontal cortex [64]. Lorcaserin is considered among several anti-obesity medicines that can treat non-alcoholic hepatic lipidosis. However, the studies carried out in this direction are insufficient and must be continued [65].

### 3.6. Bupropion

Bupropion was introduced in the US market in 1989 as an antidepressant drug. It is a weak receptor antagonist of nicotinic acetylcholine receptors and also helps in smoking cessation and seasonal affective disorders. It inhibits the reuptake of norepinephrine and dopamine neurotransmitters without inducing any changes in the levels of serotonin neurotransmission. The efficacy of bupropion is comparable with other serotonin reuptake inhibitors and antidepressants. However, bupropion is associated with side effects such as nausea, constipation, insomnia, dry mouth, headache, and dizziness [66,67]. In a randomized, double-blind, placebo-controlled trial on 50 overweight/obese women patients with BMI between 28.0 and 52.6 kg/m^2^, patients received 100 mg/d bupropion for the initial eight weeks and later switched to 200 mg twice daily. All participants were kept on a balanced diet (1600 kcal/d) and maintained food dairies. Responders continued the same treatment in a double-blind manner for an additional 16 weeks, for a total of 24 weeks. Follow-up studies showed that subjects receiving bupropion displayed a higher mean weight loss than the placebo group.

Studies in animal models have demonstrated that bupropion increased oxygen consumption in animal models via the β3-adrenoceptor and dopamine D2/D1 receptors. Moreover, more participants lost over 5% body weight than the placebo group (67% vs. 15%) [68]. The weight-reducing effects of bupropion were mainly attributed to increased thermogenesis due to increased activity of the β3-adrenergic and dopamine D2/D1 receptors. Since bupropion does not alter/reduce the food intake, the anti-obesity effects are primarily due to increased thermogenesis [69]. In September 2014, a combination of bupropion and naltrexone was approved by the FDA for obesity management. 

Naltrexone is an opioid antagonist and an approved antidepressant drug. The combination is sold under the trade name Contrave and has shown clinical safety and efficacy in clinical trials. It is an extended-release formulation, and the combination may promote weight loss by inducing satiety and increasing energy expenditure [70]. Clinical studies have demonstrated that a combination of 360 mg bupropion and 32 mg naltrexone, along with lifestyle and dietary interventions, was more effective at six months and one year than individual medicines alone. Importantly, the combination was associated with some serious side effects, thus necessitating a careful selection of patients to lower the risk of adverse events and increase the possibility of positive health outcomes [71]. For instance, patients taking a combination of naltrexone and bupropion showed side effects such as anxiety, sleep-related health issues, and depression. However, the drug combination did not increase suicidal behavior in patients [72].

Recent research has shown that the combination of naltrexone and bupropion may effectively control metabolic changes. Other studies suggest that this drug combination modulates dopaminergic expression [73]. Based on the safety and clinical efficacy analysis of drugs for treating obesity, bupropion is on the list of drugs that can effectively reduce body weight [74].

**Figure 1:**
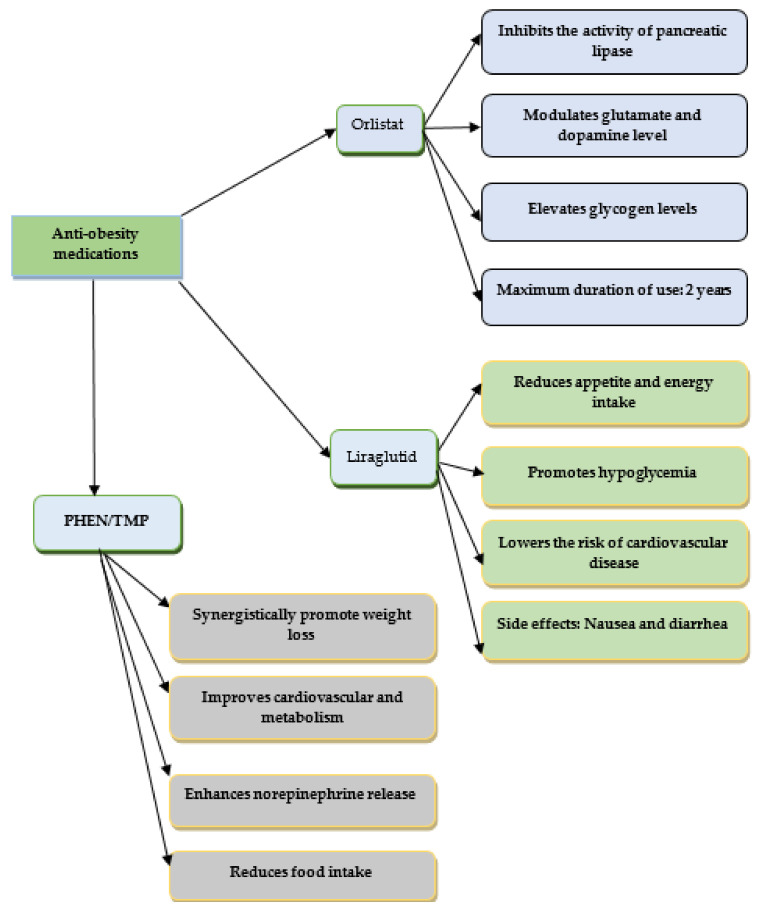
The anti-obesity effects of Orlistat, Liraglutide, and Phentermine/topiramate.

**Figure 2:**
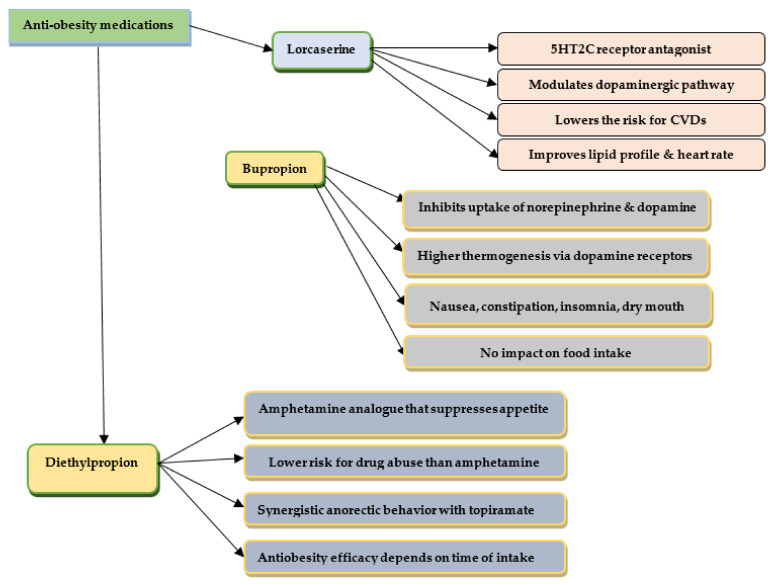
The anti-obesity effects of Lorcaserin, Bupropion, and Diethylpropion.

The anti-obesity effects of some synthetic drugs are presented in Figure 1 and Figure 2.

### 3.7. The Use of Antidiabetic Drugs and Natural Constituents in the Prevention and Treatment of Obesity

Obesity is considered the most significant risk factor for the development of type 2 diabetes [75]. In this regard, antidiabetic drugs usually have an effect on body weight control. As it is known, metformin is the first-choice therapy for type 2 diabetes [76,77]. It also has other benefits for health besides its antihyperglycemic properties. Metabolic consequences of its consumption include a reduction in hepatic gluconeogenesis and inhibition of insulin production, as well as weight loss due to the modulation of appetite regulatory centers in the hypothalamus, management of hepatic steatosis, and alteration in the gut microbiome [76]. According to Seifarth et al. [78], metformin effectively reduced weight in insulin-resistant and insulin-sensitive overweight patients. Due to its excellent safety profile, tolerability, and efficacy, it was considered first in the line of treatment of type 2 diabetes (in conjunction with modifications of the lifestyle) [79]. These promising health effects have made it an attractive opportunity for disorders associated with obesity, type 2 diabetes, and aging [76]. Zhang et al. [80] found that beinaglutide was effective in glycemic control and weight loss in treating type 2 diabetes. Recently, Gao et al. [81] found that beinaglutide was more efficient than metformin at reducing a fat mass in patients of the Chinese population who were overweight and nondiabetic. The effective daily doses of beinaglutide were in the range of 0.24–0.30 mg [82].

**Figure 3:**
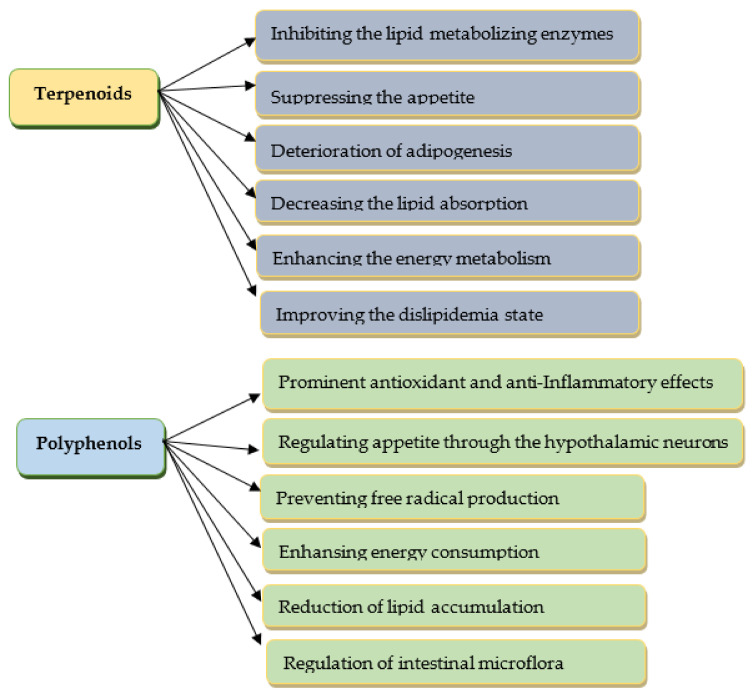
The anti-obesity effects of selected phytoconstituents.

Herbal substances are regarded as an important target for drug development because of the wide variety of phytoconstituents and their few adverse effects [83]. A huge number of bioactive compounds from medicinal plants are beneficial in obesity coping. Among the secondary metabolites of plants, mainly polyphenols and terpenoids (Figure 3), they have demonstrated effective weight management properties [83,84,85]. Some of the alkaloids have good potential in the treatment of obesity, but the significant toxicity of most of them narrows the range of their application [86].

The anti-obesity effects of phytoconstituents manifest in different ways: through inhibiting the lipid/carbohydrate-metabolizing enzymes, suppressing the appetite and adipogenesis, and inhibition of lipid absorption, as well as enhancing energy metabolism [84,87]. The modern “omics” technologies (genomics, transcriptomics, proteomics, and metabolomics) effectively evaluate the traditional healthcare phytosubstances as sources of new natural biocompounds as potential anti-obesity agents [88].

Recently, experimental research demonstrated that polyphenols as strong antioxidants were effective prebiotics in managing obesity induced by a high-fat diet [89]. As oxidative stress is crucial in the pathophysiology of obesity, modifying the concentration of inflammation mediators is associated with the number and size of adipocytes, lipogenesis, regulating appetite through the hypothalamic neurons, etc. [90]. Polyphenols could reduce body weight through different mechanisms [85,91,92]. Randomized controlled clinical trials conducted by Moorthy et al. [89] assessed the effect of polyphenols on body composition in the overweight, obese population. These studies showed some decrease in body weight by a mean of 1.47 ± 0.58 kg. Additionally, polyphenols could effectively prevent increases in weight [89]. The polyphenol-rich extract of the *Vaccinium corymbosum* leaves modified with arginine demonstrated its effectiveness in managing the metabolic syndrome [93]. Suzuki et al. [94] found a positive effect of black and green tea catechins on obesity. Quercetin and other flavonoids with antioxidant, anti-inflammatory, and hepatoprotective effects can effectively prevent metabolic diseases [95,96,97]. The intake of flavonoids can reduce the risk of metabolic syndrome disorders and rare side effects [95].

Nani et al. [98] concluded that the overproduction of reactive oxygen species is associated with the inflammatory process in obesity mediated through nuclear factor-κB. Chen et al. [99] reported that polyphenols could enhance the energy consumption and weight loss due to increased fat oxidation. Polyphenols are regarded as being very effective in the inactivation of reactive oxidant species [100]. Polyphenolic compounds from fruits and vegetables reduce lipid accumulation and enhance intestinal microflora [99]. The fruits and leaves of some *Ericaceae* species (*Vaccinium corymbosum, Vaccinium myrtillus,* etc.) [93,101,102] possess significant lipid-lowering properties and anti-obesity potential due to the presence of valuable phenolic compounds. Polyphenols of marine algae can transform problematic ‘white’ adipose tissue into ‘brown’ (rich in mitochondria) and, in this way, enhance energy consumption [103].

In the last decade, several researchers [104,105] have investigated the therapeutic effect of *Ginkgo biloba* extract in treating obesity and related disorders. The long-term therapy using an excerpt from *Ginkgo biloba* leaves showed an anti-obesogenic effect on rats [104]. Thomaz et al. [105] revealed that *Ginkgo biloba* extract mainly consists of flavonoids (25.21%). The chromatographic analysis revealed that flavonoids such as quercetin, kaempferol, rutin, and isorhamnetin were its predominant components [105].

The discovery of leptin at the end of the 20th century created hopes for an effective treatment of obesity, as this peptide hormone effectively regulates the body mass and lipolysis [106]. However, the development of resistance to the leptin influence, which is characterized by the overconsumption of nutrients due to reduced satiety, has been a big obstacle [107]. Liu et al. [107] discovered that the pentacyclic triterpene celastrol isolated from the roots of *Tripterygium wilfordi* possesses a significant anti-obesity effect as a leptin sensitizer. It can suppress food intake and causes up to 45% weight loss in obese mice by increasing the leptin sensitivity. In addition to the ability to regulate leptin sensitivity and lipid metabolism, it also positively influences the gut microbiota [108].

The anti-obesity potential of triterpene saponins was elucidated by Marrelli et al. [109]. Saponins can modulate adipogenesis and appetite and inhibit pancreatic lipase [109]. Alkaloid capsaicin, an active component of chili peppers, demonstrated great anti-obesity potency [110]. For decades, natural guanidine-containing phytosubstances from the French lilac (*Galega officinalis*) were used for their antidiabetic, hypolipidemic, and antiaging effects [76].

The weight management effect of carotenoids was found by Mounien et al. [111]. Gammone and D’Orazio [112] found the anti-obesity effect of fucoxanthin (carotenoid from marine algae). Carotenoid lycopene, which accumulates significantly in ripe tomatoes, demonstrated protection against diabetes and obesity [113]. Bjørklund et al. [114] revealed the ability of the other carotenoid astaxanthin, synthesized by numerous microalgae, yeasts, and bacteria, to manage the overweight outcome. Radice et al. [115] found that supplementation of the experimental animals with astaxanthin had positive effects on a variety of symptoms associated with obesity through the hypoglycemic and lipid-lowering capacity, as well as mitigating the immune system. Calanus oil, a natural product from marine crustacean *Calanus finmarchicus,* which is rich in astaxanthin, has a noticeable effect in treating low-grade inflammation related to obesity [116].

It should be noted that seaweeds were regarded as promising sources of various anti-obesity agents, such as phlorotannins, alginates, fucoxanthin, and fucoidans [87]. Fucoxanthin and fucoidans could inhibit lipid absorption and metabolism, as well as the differentiation of adipocytes, alginates reduce the feeling of hunger, and polyphenol phlorotannin possesses significant antioxidant and anti-inflammatory properties [87].

Cannabidiol from *Cannabis sativa,* which is widely known for its neurological effects, also has been considered an anti-inflammatory, antitumor, and anti-obesity agent [117]. As cannabinoid receptors regulate food consumption, thermogenesis, and inflammation, the intake of cannabinoids could help to reduce food intake and body weight [118].

Recently, De Blasio et al. [119] demonstrated that essential oils as multicomponent mixtures of volatile terpenoids and other bioactive compounds promote the decrease of fat mass and exert a positive weight management effect. It should be mentioned that essential oils exert these health-promoting effects when inhaled or taken with the diet [119]. Artemisinin, a sesquiterpenoid from the *Artemisia annua*, is a famous antimalarial drug [120]. In addition to the anti-parasite activity, artemisinin has also displayed antitumor, anti-inflammatory, and anti-obesity properties. Its anti-inflammatory and immunoregulatory effects are valuable in obesity coping, since chronic inflammation is implicated in the pathogenesis of metabolic disorders [116,120]. Islam et al. [121] summarized that several diterpenoids exert anti-obesity effects through various mechanisms. Among them, carnosol, carnosic acid, steviol, and andrographolide could be examples of effective weight management agents.

Experimental evidence was obtained of the anti-obesity effects of *Ananas comosus* juice [122] and papain (proteolytic enzyme) from *Carica papaya* fruits [123]. The anti-obesity properties of sulforaphane from broccoli (*Brassica oleracea* var. *italica)* were revealed by Ranaweera et al. [124].

Saffron (*Crocus sativus*) stigmas are famous spices and a promising natural antioxidant, anticancer, and anti-obesity phytosubstance [125,126]. Aromatic compound safranal and some carotenoids (crocin and picrocrocin) are regarded as the main bioactive constituents of *Crocus sativus* stigmas [126].

As it is known, many health disorders, such as diabetes, chronic inflammatory diseases, and obesity, are associated with uncontrolled sugar consumption [127]. As an artificial sweetener, Xylitol effectively prevents metabolic syndrome and obesity [127,128]. It can reduce the increased blood glucose level, body weight, and other unhealthy syndromes [127].

Some vitamins also possess substantial anti-obesity potential [6,129]. The antioxidant and hepatoprotective activity of tocopherol helps in preventing metabolic syndrome [130]. A deficiency of some vitamins in the body can cause excess weight. Thus, Thomas-Valdés et al. [6] concluded that most vitamins were deficient in obese persons, especially the fat-soluble vitamins, vitamin B12, folic acid, and ascorbic acid.

## 4. Limits in the Pharmacological Treatment of Obesity

Although several anti-obesity treatment options are available, the use of pharmacological intervention to combat obesity has several limitations. For instance, most anti-obesity drugs target satiety signaling in the brain. However, the long-term use of synthetic medications that target satiety signaling is unsafe and causes chronic disorders and side effects. This limitation suggests that novel anti-obesity treatment options must focus on signaling pathways that increase energy expenditure and create a negative energy balance [131]. According to an estimate, 25 anti-obesity medications were withdrawn from the market between 1964 and 2009. Importantly, 23 of them were targeted against monoamine neurotransmitters. Anti-obesity medications were associated with psychiatric disorders, cardiotoxicity, and drug dependence. Thus, anti-obesity drugs that target neurotransmitters raise serious safety concerns, and greater transparency and scrutiny in clinical trials are warranted before a drug is approved for clinical use [132]. According to an observation, the weight loss induced by most of the anti-obesity drugs is only <4 kg compared to the control group. However, the adverse effects associated with long-term use do not justify their usefulness in obesity management. Moreover, most anti-obesity medications are suggested as adjunct therapy to lifestyle and dietary interventions. Several obese patients do not respond well to anti-obesity medications; hence, no significant weight loss (>5%) is observed. In non-responding patients, discontinuing anti-obesity medication helps reduce safety concerns and treatment costs. Therefore, there is an urgent need to develop novel and effective anti-obesity drugs that can induce more weight loss with the least side effects. This also requires the study and discovery of novel pathways and new molecular targets so that the obesity pandemic can be handled effectively [133,134]. A randomized clinical trial’s findings showed that most anti-obesity drugs demonstrated an average weight loss between 3% and 9% after 1-year treatment compared with the placebo group. However, enough data related to race, ethnicity, and gender are unavailable. Additionally, a high drop-out rate observed in anti-obesity clinical trials is another major limitation and prevents the generalization of clinical findings. It has been observed that anti-obesity medications only lower the glycemia and do not cause a significant reduction in the lipid profile and blood pressure. Finally, limited studies are available to address the safety issue of anti-obesity drugs in children and patients taking medications post-bariatric surgery [135]. Concentrating efforts to fill in the knowledge gap is necessary to increase the drug efficacy and improve the safety profile of anti-obesity medications [136].

Pharmacotherapy is an important tool in the fight against obesity. Still, very few drugs are currently approved for the treatment of obesity due to several limiting factors, including possible toxic effects on the patient’s body. This fact encourages the active development of alternative pharmaceutical drugs against obesity. Effective pharmacotherapy can only be after a deep analysis of the pathogenesis of obesity [137]. Special attention should be paid to developing specific guidelines for the dosage of radiopharmaceuticals. This task remains relevant due to the negative effects of an incorrect dosage, including toxicity due to the high radioactivity of such drugs. The need for such studies is due to the increase in obese people among the Western population [138]. The potential side effects of pharmacotherapy and surgical methods in treating obesity prompt the search for other ways to treat this pathology. One of these methods is electroacupuncture, during which electrical stimulation is transmitted through acupuncture needles to the body. The authors of the studies provided evidence of the effectiveness of the use of electroacupuncture in the treatment of obesity [139]. The study authors reported that synthetic drugs are gaining ground in anti-obesity therapy. This fact should cause concern, since such drugs’ positive effects and safety have not been studied enough. In particular, the study results for anti-obesity medicines Aplex and Venera indicate their negative side effects on the kidneys and liver, confirmed by the physiological and biochemical parameters [140].

The prevalence of obesity among children and adolescents remains an important problem that complicates the reluctance of many adolescents to change their lifestyles. The European Medicines Agency (EMA) for a long time did not approve pharmacological drugs for the treatment of obesity in childhood and only in 2021 allowed the use of Liraglutide for the treatment of obesity in persons aged 12–17 years. In the study, the body mass index decreased by 5% after 56 weeks in 43.3% of participants in the Liraglutide group [141]. The study of the pathophysiological mechanisms of obesity was the basis for creating new strategies for treating this disease. Currently, the number of anti-obesity pharmaceuticals remains insignificant, so there is still a need to develop new anti-obesity drugs with a high safety profile and clinical efficacy. Further analysis of the pathophysiological mechanisms of the development of obesity will contribute to a personalized approach to the treatment of obesity and the safe achievement of a sustainable weight [142].

## 5. Conclusions

Obesity is a chronic metabolic complication, and its management requires long-term medication, lifestyle modifications, and dietary interventions. Patients taking anti-obesity medications may suffer from side effects such as psychiatric disorders, anxiety, depression, and vitamin deficiency. As several anti-obesity medications have been withdrawn in the past owing to serious side effects, the discovery of new metabolic pathways has opened up many opportunities for new drug molecules that may have lesser imperfections and more therapeutic efficacy. 

The current knowledge of the available anti-obesity medicines that have synthetic or natural origins, their mechanisms of action, and their possible shortcomings was summarized in the study. Various classes of natural compounds are promising agents to combat the obesity pandemic. Developing safer drugs may require polytherapeutic strategies to combat the global obesity pandemic.

## Data Availability

Not applicable.
